# Deleting *qseC* downregulates virulence and promotes cross-protection in *Pasteurella multocida*

**DOI:** 10.1186/s13567-021-01009-6

**Published:** 2021-11-20

**Authors:** Yang Yang, Pei Hu, Lixu Gao, Xiang Yuan, Philip R. Hardwidge, Tian Li, Pan Li, Fang He, Yuanyi Peng, Nengzhang Li

**Affiliations:** 1 College of Veterinary Medicine, Southwest University, Chongqing, 400716 China; 2 College of Veterinary Medicine, Kansas State University, Manhattan, KS USA

**Keywords:** Quorum sensing gene, Deletion, Virulence, Vaccine, Cross-protection

## Abstract

QseC, a histidine sensor kinase of the QseBC two-component system, acts as a global regulator of bacterial stress resistance, biofilm formation, and virulence. The function of QseC in some bacteria is well understood, but not in *Pasteurella multocida*. We found that deleting *qseC* in *P. multocida* serotype A:L3 significantly down-regulated bacterial virulence. The mutant had significantly reduced capsule production but increased resistance to oxidative stress and osmotic pressure. Deleting *qseC* led to a significant increase in *qseB* expression. Transcriptome sequencing analysis showed that 1245 genes were regulated by *qseC*, primarily those genes involved in capsule and LPS biosynthesis and export, biofilm formation, and iron uptake/utilization, as well as several immuno-protection related genes including *ompA*, *ptfA*, *plpB*, *vacJ*, and *sodA*. In addition to presenting strong immune protection against *P. multocida* serotypes A:L1 and A:L3 infection, live Δ*qseC* also exhibited protection against *P. multocida* serotype B:L2 and serotype F:L3 infection in a mouse model. The results indicate that QseC regulates capsular production and virulence in *P. multocida*. Furthermore, the *qseC* mutant can be used as an attenuated vaccine against *P. multocida* strains of multiple serotypes.

**Supplementary Information:**

The online version contains supplementary material available at 10.1186/s13567-021-01009-6.

## Introduction

*Pasteurella multocida* is a pathogenic bacterium that causes a variety of diseases in livestock, poultry, and humans [1]. It can be classified into five capsular serotypes (A, B, D, E, F) based on capsular polysaccharides [2], and eight genotypes (L1-L8) based on the lipopolysaccharide outer core biosynthesis locus [3, 4]. Various environmental stresses in animals lead to an increase of pasteurellosis caused by *P. multocida*, which results in significant economic losses. At present, most commercial vaccines for *P. multocida* are focused on controlling a specific serotype and a universal vaccine against multiple serotypes is lacking.

Quorum sensing (QS) is a bacterial communication system that controls a variety of physiological functions of bacteria including nutrient uptake, biofilm formation, and virulence factor expression [5]. Bacterial quorum sensing systems are divided into four categories: quorum sensing system mediated by acyl-homoserine lactone (AHL), signal peptide, Autoinducers 2 (AI-2), and Autoinducers 3 (AI-3) [6, 7]. Autoinducers (AIs) are secreted and recognized by bacterial quorum sensing system receptors to initiate signal transduction processes after AI concentrations reach a critical threshold [8, 9]. QS inhibitors can be used to downregulate virulence, disrupt bacterial communication, and attenuate infection symptoms [10, 11].

QseC belongs to the QseBC quorum sensing system mediated by AI-3. AI-3 is first recognized by QseC and induces QseC auto-phosphorylation. QseC then dephosphorylates QseB, and then the dephosphorylated QseB activates the expression of specific genes [12, 13]. In addition to activating QseB, QseC can also activate QseF and KdpE, which regulate the expression of virulence- and flagella-related genes [14–16]. Loss of QseC function leads to reduced resistance to environmental stress and to reduced virulence [17–19].

The QseBC quorum sensing system is present in all *P. multocida* serotypes, but its function in this organism is still unclear. Cross-protection can be promoted by an *aroA* gene deletion in some *P. multocida* strains [20]. A *Shigella hfq* gene mutant provided cross-protection against *Shigella* strains of broad serotype [21]. In this study, we investigated the role of QseC in *P. multocida* virulence. Our data support the role of QseC as a regulator of capsule production, virulence, biofilm formation, stress resistance, and show that QseC can negatively regulate the expression of cross-protective antigens in *P. multocida*. A *qseC* deletion strains provides robust cross-protection against multiple *P. multocida* serotypes.

## Materials and methods

### Bacterial strains, plasmids and cultural conditions

Bovine *P. multocida* capsular serotype A strain CQ2 (A:L3, PmCQ2) was isolated from the lung of a dead calf that suffered from pneumonia in Chongqing, China. Bovine *P. multocida* capsular serotype A strain CQ1 (A:L3, PmCQ1), CQ4 (A:L3, PmCQ4), and CQ5 (A:L3, PmCQ5) were isolated from the nasal swabs of calves in Chongqing, China. Bovine *P. multocida* capsular serotype F strain F (F:L3, PmF) was isolated from the lung of a dead calf that suffered from pneumonia in Mianyang city, Sichuan province, China. Bovine *P. multocida* capsular serotype B strain B (B:L2, PmB) and porcine *P. multocida* capsular serotype A strain CVCC1662 (A:L1, PmP) were purchased from the China Institute of Veterinary Drug Control. Rabbit *P. multocida* capsular serotype A strain R (A:L3, PmR) was isolated from the liver of a dead rabbit in Chongqing, China. Avian *P. multocida* capsular serotype A strain Q (A:L1, PmQ) was isolated from a dead duck in Chongqing, China. Strains were streaked on Martin medium agar plates (Qingdao Hope Bio-Technology Co., Ltd., Qingdao, China), and incubated at 37 °C for 24 h. One colony of each strain was inoculated into 5 mL Martin broth and cultured for 12 h at 37 °C with shaking at 220 rpm. E. *coli* DH5α competent cells were purchased from Beijing Dingguo Changsheng Biotechnology CO.LTD. Plasmid pUC19oriKan^R^ for mutant construction in *P. multocida* was constructed in our previous study [22]. *E*. *coli* DH5α recombinant strains were screened on plates of Luria–Bertani (LB) medium supplemented with 100 μg/mL kanamycin. The pMc-Express plasmid was obtained from Dr Sanjie Cao, Sichuan Agricultural University.

### Mice

All mouse use was approved by the Laboratory Animal Ethics Committee of Southwest University (Permit number: IACUC-20200803-01). Kunming mice (Female, 6–8-week-old) were purchased from the Laboratory Animal Center, Chongqing Medical University, Chongqing, China, and mice were fed in IVC system (Individual Ventilated Cages; Suzhou Xinqu Fengqiao Experimental Animal Cage) with free access to water and food under controlled temperature (26 °C).

### Phylogenetic analysis of QseC in *P. multocida*

QseC sequences from *P. multocida*, *E. coli*, *Pasteurella canis*, *Pasteurella dagmatis*, *Pasteurella oralis*, and *Salmonella enterica* et al. in the NCBI Database were downloaded. Four QseC sequences from *P. multocida* capsular serotype A, B, D, and F (CQ2, B, HN06, F) were aligned pairwise by using NCBI BLAST, and the neighbor-joining phylogenetic analysis of QseC was performed by using MAGA-X software and the circular tree of QseC was made on the website.

### Construction of marker-free mutant and complementary strain

Primers used in mutant and complementary strain construction are listed in Table 1. PmCQ2 genomic DNA was extracted by using a bacterial genomic DNA extraction kit (DP302, Tiangen). A 350 bp upstream homologous recombination arm (Up-arm) and a 350 bp downstream homologous recombination arm (Down-arm) of the *qseC* gene were amplified by PCR, and the PCR fragments were purified using a DNA gel extraction kit (D0056, Beyotime). Up- and Down-arm fragments were linked by using overlap PCR, and then inserted into the H*ind* III and B*amH* I sites of pUC19oriKan^R^ using In-Fusion® HD Cloning Kit (PT5162-1, Clontech) to generate the recombinant plasmid pUC19oriKan^R^-qseC_up+down_. Subsequently, the plasmid pUC19oriKan^R^-qseC_up+down_ was transferred into PmCQ2 competent cell by electroporation and the Δ*qseC* mutant was selected on Martin agar plate and verified by using PCR. The mutant was serially passaged for 30 generations for genetic stability analysis prior to use. A *qseC* complementation strain was constructed by using a recombinant gene expression plasmid. Briefly, the *egfp* gene expression cassette fragment was amplified from plasmid pMc-Express, and inserted into the H*ind* III and B*amH* I sites of pUC19oriKan^R^ to generate recombinant plasmid pUC19oriKan^R^-egfp. The *egfp* gene was replaced with the *qseC* gene at the S*al* I and X*ba* I sites of pUC19oriKan^R^-egfp to generate complementation plasmid pUC19oriKan^R^-com*qseC* and then introduced into *P. multocida* by using electroporation to generate *qseC* gene complementary strain C-*qseC*.

**Table 1:** **PCR primers.**

Primer	Sequence(5’-3’)	Product size (bp)
*qseC* 5’Arm-F	GACCATGATTACGCCAAGCTTTGATTGCACGTTTGCAGGCA	350
*qseC* 5’Arm-R	TTTTATATAATTAAGCCATTTCATCATTTT	
*qseC* 3’Arm-F	AATGGCTTAATTATATAAAAGGATTTAGAT	350
qseC 3’Arm-R	TTTATCGGTACCCGGGGATCCAGCGAGATTATTCTACACCG	
d*qseC*-F	GCAAACAAGTTTACGAGTTC	1300
d*qseC*-R	CACATTTTCTAACCGGAATTG	
pUC19-F	GAGCGGATAACAATTTCACAC	151
pUC19-R	ATTTAAGAATACCTTGCCGC	
*KMT1*-F	ATCCGCTATTTACCCAGTGG	460
*KMT1*-R	GCTGTAAACGAACTCGCCAC	
*egfp*-F	GACCATGATTACGCCAAGCTTCCGCGCCAACCGATAAAACC	1111
*egfp*-R	TTTATCGGTACCCGGGGATCCCAATTCGCCCTATAGTGAGT	
com*qseC*-F	CTAGTGAATTCTGCAGTCGACATGAAATGGCTTAAGCAAAC	1374
com*qseC*-R	TGGCCGTCGTTTTACTCTAGATTAAATTTTATTTAATAAAA	

### Quantification of capsular polysaccharide

The capsular polysaccharides were measured by using a method as previously described [23]. Briefly, three to four colonies were randomly selected from each strain (PmCQ2, Δ*qseC* and C-*qseC*), and then inoculated in fresh Martin broth medium and incubated at 37 °C for 8 h with shaking at 200 rpm. The bacterial cultures were centrifuged at 13 200 rpm for 15 min and bacterial pellets were washed twice with PBS and re-suspended in 1 mL PBS. Suspensions were incubated at 42 °C for 1 h, and then centrifuged at 13 200 rpm for 10 min, after which the supernatants were collected. The supernatant (10 μL) of each sample was added to 90 μL capsule staining solution (0.2 mg/mL Stains all, 0.06% glacial acetic acid in 50% formamide), mixed together, and then assayed by measuring absorbance at 640 nm. The standard curve was made by using a hyaluronic acid standard.

### Growth curve analysis

Three to four colonies randomly selected from each strain (PmCQ2, Δ*qseC* and C-*qseC*) were inoculated in 5 ml of Martin broth medium and incubated at 37 °C for 10 h with shaking at 200 rpm. The bacterial cultures were sub-cultured at a dilution of 1:100 into fresh Martin broth medium, respectively, and incubated at 37 °C with shaking at 200 rpm. Cultures were sampled at every 1.5 h after incubation, and the bacterial concentrations (OD_600_) at each time-point were measured.

### Biofilm quantification

Biofilm formation ability was measured by using a crystal violet staining method as previously described [24]. Briefly, overnight bacterial cultures of PmCQ2, Δ*qseC* and C-*qseC* were diluted with BHI broth medium (1 × 10^8^ CFU/mL), added to 48-well cell culture plates with 400 μL/well, centrifuged at 3800 rpm for 10 min, the negative control was BHI broth with 400 μL/well, incubated at 37 °C for 48 h, respectively. The bacterial culture in each well was drawn off with a syringe. Methanol (200 μL/well) was added into each well for 30 min, then washed 3 times with PBS and dried at room temperature for 1 h. Crystal violet (1%, 200 μL/well) was added into each well for 30 min at 37 °C, then washed 3 times with PBS, and dried at room temperature. Glacial acetic acid (33%, 200 μL/well) was added into each well to dissolve the Crystal violet from the stained biofilm for 30 min at 37 °C. The dissolved crystal violet sample in each well was assayed by measuring absorbance at 630 nm.

### Stress resistance assay

The stress-resistances of PmCQ2, Δ*qseC* and C-*qseC* were investigated using methods as previously described [19]. For oxidative stress assays, bacterial cells were treated with 5 mM, 10 mM or 20 mM H_2_O_2_ for 1 h at 37 °C. For hyperosmotic tolerance assays, bacterial cells were treated with 100, 200 or 300 mM NaCl at 37 °C for 1 h. The stress resistances were calculated as the formula (stress-treated bacteria CFU mL^−1^/ control bacteria CFU mL^−1^) × 100. In each test, there were three biological repeats and all experiments were conducted independently three times.

### Pathogenicity of wild type, mutant and complementary strain

Kunming mice (Female, 6–8-week-old) were divided into three groups and intraperitoneally infected with PmCQ2, Δ*qseC* and C-*qseC* (3.48 × 10^5^ CFU), respectively. Ten infected-mice in each group were monitored for 7 days for the survival rate calculation. After infection, mice with serious clinical signs (low energy, slow reaction, no eating and drinking, eyes closed and faint breathing) were considered moribund and were euthanized by cervical dislocation. At 8, 16, and 24 h after infection, six mice at each time point were euthanized by cervical dislocation and the lungs were collected for the measurements of bacterial loads. Lungs were also collected at 24 hpi for histopathological examination.

### Median lethal dose (LD_50_) measurement

Female Kunming mice (6–8-week-old) were randomly divided into 8 groups (*n* = 10/group). Four groups of mice were intraperitoneally infected with 100 µL Δ*qseC* (dose ranging from 4.46 × 10^5^ to 3.72 × 10^8^ CFU), and other 4 groups of mice were intraperitoneally infected with 100 µL C-*qseC* (3.8 × 10^2^, 3.8 × 10^3^, 3.8 × 10^4^, 3.8 × 10^6^ CFU). The mice were monitored for 7 days after infection with mice with serious clinical signs (low energy, slow reaction, no eating and drinking, eyes closed, and faint breathing) were considered moribund and were euthanized by cervical dislocation. The number of dead mice was recorded daily, and the LD_50_ determinations were done by using the Bliss method.

### Immune protection assay

Kunming mice (Female, 6–8-week-old) were randomly divided into 45 groups (*n* = 10 mice/group). The immunization and challenge schedule are listed in Table 2. At day-21 after inoculation, the bloods were collected via the tail vein route, kept at 37 °C for 20 min and then 4 °C overnight for serum separation. Mice were intramuscularly injected with 3.8 × 10^7^ CFU PmCQ1(intramuscular route: LD_50_ = 3.8 × 10^2^ CFU), 4.8 × 10^7^ CFU PmCQ2 (intramuscular route: LD_50_ = 3.4 × 10^3^ CFU) [22], 3.6 × 10^7^ CFU PmCQ4 (intramuscular route: LD_50_ = 2.1 × 10^3^ CFU), 4.5 × 10^7^ CFU PmCQ5 (intramuscular route: LD_50_ = 4.5 × 10^3^ CFU), 1.0 × 10^7^ CFU PmB (intramuscular route: LD_50_ = 5.0 × 10^3^ CFU), 2.0 × 10^8^ CFU PmF (intramuscular route: LD_50_ = 1.0 × 10^8^ CFU), 10 CFU PmP (intramuscular route: LD_50_ ≈ 1 CFU), 10 CFU PmQ (intramuscular route: LD_50_ ≈ 1 CFU), and 1.0 × 10^6^ CFU PmR (intramuscular route: LD_50_ = 1.0 × 10^4^ CFU), respectively. The mice were monitored for 7 days after infection. Mice with serious clinical signs (low energy, slow reaction, no eating and drinking, eyes closed and faint breathing) were considered moribund and were euthanized by cervical dislocation. The number of dead mice was recorded daily, and the protection rates were calculated based on final survival numbers of mice in each group.

**Table 2:** **Immunization schedule and challenge.**

Groups	Bacterin (CFU/mL)	Primary immune dose (mL)	Booster dose (mL)	Booster day (dpi)	Challenge strain	Challenge day (dpi)
Live Δ*qseC*	4.5 × 10^7^	0.1			PmCQ1, PmCQ2, PmCQ4, PmCQ5, PmB, PmF, PmP, PmQ, and PmR, respectively	21
PBS		0.1			PmCQ1, PmCQ2, PmCQ4, PmCQ5, PmB, PmF, PmP, PmQ, and PmR, respectively	21
Inactivated PmCQ2	5.0 × 10^9^	0.2	0.1	7	PmCQ1, PmCQ2, PmCQ4, PmCQ5, PmB, PmF, PmP, PmQ, and PmR, respectively	21
Inactivated Δ*qseC*	5.0 × 10^9^	0.2	0.1	7	PmCQ1, PmCQ2, PmCQ4, PmCQ5, PmB, PmF, PmP, PmQ, and PmR, respectively	21
PBS emulsifier		0.2	0.1	7	PmCQ1, PmCQ2, PmCQ4, PmCQ5, PmB, PmF, PmP, PmQ, and PmR, respectively	21

*dpi* day after primary immunization.

### Antibody titer determination

Total bacterial cell proteins of each strain were prepared by using ultrasound pyrolysis and the proteins concentration was measured by using the Bradford method. Each well of 96-well ELISA plates was coated with 1 μg protein in 100 μL carbonate buffer (0.05 M, pH9.0) at 4 °C overnight. The next day, the plates were washed 5 times with PBST (PBS containing 0.05% Tween-20), and then treated with blocking buffer (5% skim milk in PBST) at 37 °C for 1 h. Next, the plates were washed 5 times with PBST. The sera were serially diluted in twofold increments in 96-well plates and incubated at 37 °C for 1 h. The sera from non-immunized mice served as negative controls. After washing, 100 μL of HRP-conjugated goat anti-mouse IgG (H + L) antibody (Sigma; diluted at 1: 10 000) was added and incubated for 1 h at 37 °C, followed by washing. Then, 100 μL of TMB were added for 10 min (Beyotime biotechnology, China) and stopped by the addition of 2 M H_2_SO_4_, before the absorbance quantification at OD_450_ was done [25]. When the ratio of the positive value (P) of the maximum dilution multiple sera of immunized mice to the negative value (N) of sera of non-immunized mice is greater than 2.1 (P/N > 2.1), the maximum dilution ratio is the serum antibody titer.

### Transcriptome analysis

One milliliter of mid-logarithmic phase of PmCQ2 and Δ*qseC* were seeded in 100 mL Martin broth, respectively, incubated at 37 °C for 6 h with shaking at 200 rpm. The bacterial cultures were centrifuged at 6000 rpm for 10 min at 4 °C. Pellets were washed three times with ice-cold PBS, frozen in liquid nitrogen, and sent to Shanghai Personalbio Technology Co., Ltd. for transcriptome sequencing and analysis (HiSeq, Illumina).

### qRT-PCR

Total bacterial RNA was extracted using an RNAprep pure Animal/Cell/Bacteria Kit (TIANGEN, China). RNA concentration was normalized among samples and cDNA synthesis was performed in 20 μL by using an iScript cDNA synthesis kit (Bio-Rad, USA). Quantitative real-time RT-PCR was performed according to a previous study [26] using a CFX96 instrument (Bio-Rad, USA). Primers used in qRT-PCR are listed in Additional file 1.

### Statistical analysis

Statistical analyses were performed using GraphPad Prism 6.0 software. Mouse survival rates were evaluated using Kaplan–Meier analysis, while other data analyses were performed using unpaired *t*-tests. Data were expressed as means ± SD, and *P* < 0.05 was considered significant.

## Results

### Phylogenetic analysis of QseC in *Pasteurella multocida*

We observed that *qseC* is present in 264 of the 288 genome-sequenced *P. multocida* strains (no capsular serotype E strain has been sequenced). To determine the similarity of QseC sequences in *P. multocida* strains, four QseC sequences from *P. multocida* capsular serotype A, B, D, and F (CQ2, B, HN06, F) were aligned pairwise by using NCBI BLAST, revealing high identities among QseC sequences in *P. multocida* serotypes (≥ 98.91% conservation). The phylogenetic analysis of QseC performed by using MEGA-X revealed that QseC from 264 *P. multocida* belonged to a similar evolutionary branch as compared with that from other bacterial species (Additional file 2).

### Construction and characterization of marker-free *qseC* mutant

**Figure 1:**
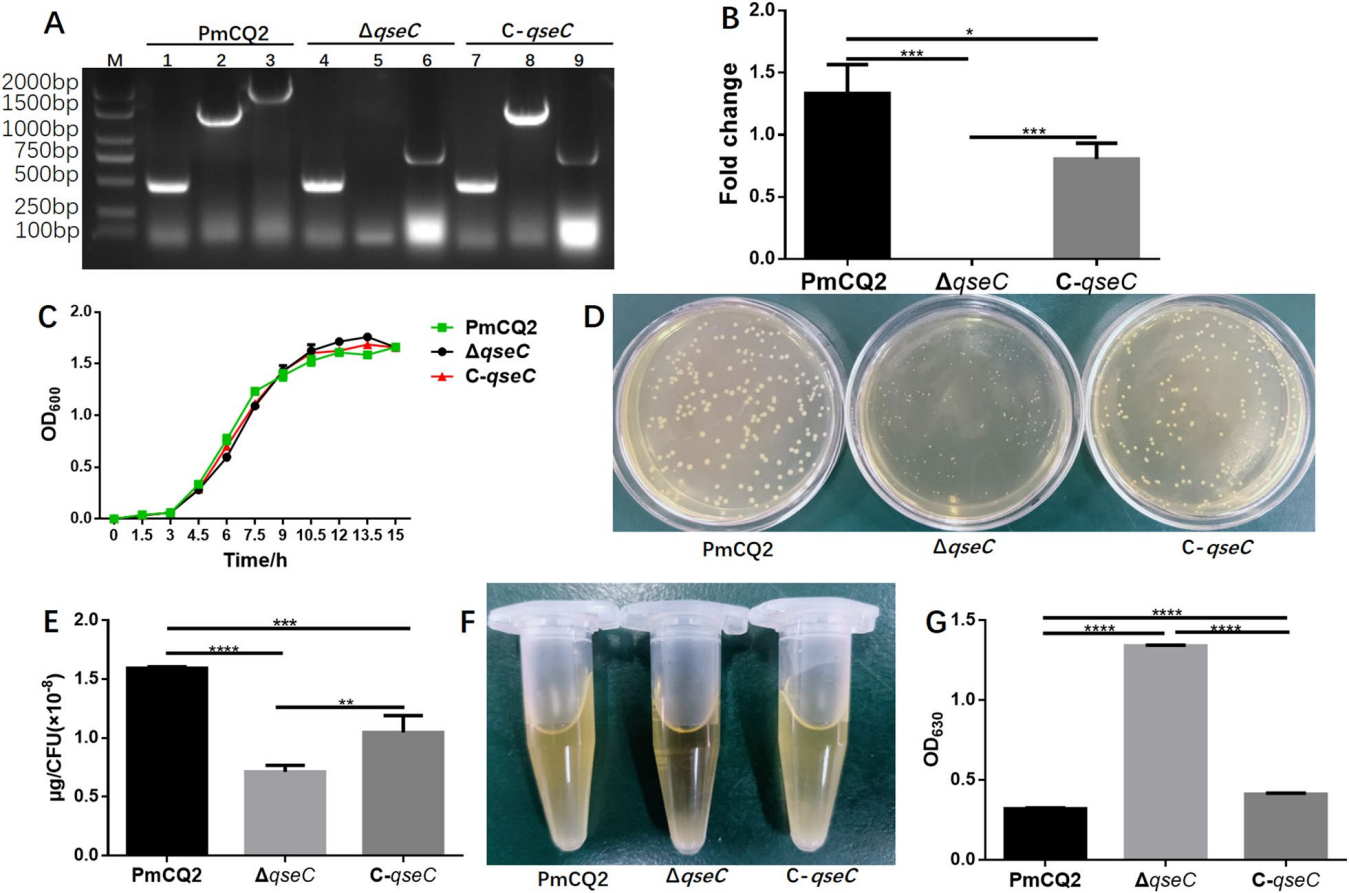
**Construction and characterization of the *qseC* mutant and complementary strain C-*qseC***. **A** PCR confirmation of mutant and complementation strains. M: DNA marker; Lanes1-3: PmCQ2; Lanes 4–6: Δ*qseC*; Lanes 7–9: C-*qseC*. Lanes 1, 4, and 7: *P. multocida* detection by using species-specific primers KMT1-F/KMT1-R (positive control); Lanes 2, 5, and 8: *qseC* gene detection by using primers com*qseC*-F /com*qseC*-R; Lanes 3, 6, and 9: *qseC* gene detection by using primers *qseC*5'Arm-F/*qseC*3'Arm-R. **B** *qseC* gene transcripts measured by using qRT-PCR. **C** Growth curves of strains cultured in Martin broth media at 37 °C with shaking at 200 rpm. **D** Colony morphology of PmCQ2, Δ*qseC* and C-*qseC* cultured on Martin agar plates. **E** Capsular polysaccharide production. **F** The centrifugally packed state of bacterial cells centrifuged at 10 000 rpm for 5 min. **G** Quantification of biofilm production. Panels **B, C**: values are expressed as the mean ± SD, *n* = 3. Panels **E, G**: values are expressed as the mean ± SD, *n* = 5. * *P* < 0.05; ** *P* < 0.01; *** *P* < 0.001; **** *P* < 0.0001; ns means not significant.

**Figure 2:**
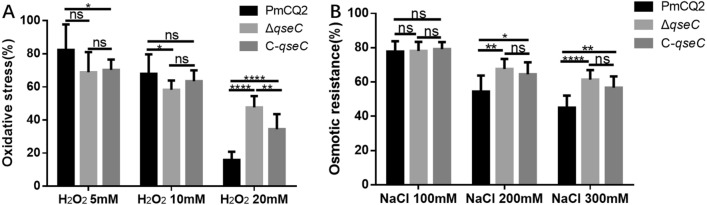
**Stress tolerance analyses**. **A** The bacterial cells were treated with 5 mM, 10 mM, or 20 mM H_2_O_2_ for 1 h at 37 °C. **B** The bacterial cells were exposed to 100 mM, 200 mM and 300 mM NaCl for 1 h at 37 °C. Values are expressed as the mean ± SD, *n* = 9. * *P* < 0.05; ** *P* < 0.01; *** *P* < 0.001; **** *P* < 0.0001; ns means not significant.

To investigate the role of QseC in *P. multocida,* we constructed a marker-free *qseC* mutant (Δ*qseC*) in the bovine *P. multocida* capsular serotype A strain CQ2, and a *qseC* gene complementary strain C-*qseC* derived from *qseC* mutant. PCR amplification showed that the *qseC* gene was not in the chromosome of Δ*qseC* and C-*qseC*, but exists in a plasmid in C-*qseC* (Figure 1A). To further confirm the mutant and complementary strain, RT-PCR was conducted and the results showed that the *qseC* gene transcripts only exist in the wild type and complementary strain but not in the mutant (Figure 1B). Δ*qseC* was stable for more than 30 passages (data not shown), and the growth curve was similar to the WT strain (Figure 1C). The colony morphology of Δ*qseC* was far smaller than that of PmCQ2 and C-*qseC* (Figure 1D). Considering the similar growth rates among the three strains and the abundant capsular polysaccharide in PmCQ2, the colony size of mutant maybe related to the decrease of the capsular polysaccharide content in cells. The capsular polysaccharides extracted from PmCQ2, Δ*qseC* and C-*qseC* were detected. Capsular polysaccharide production was significantly reduction in Δ*qseC* compared with wild type, and followed by C-*qseC* (Figure 1E). As capsular polysaccharide content also affects the centrifugation status of bacteria, Δ*qseC* cells were more easy centrifuged to the bottom of tube with clear transparent supernatant (Figure 1F). Capsular polysaccharide production trends in *P. multocida* capsular serotype A are in opposition to the propensity of strains to produce biofilms [27]. Biofilm quantification detection showed that the biofilm formation in Δ*qseC* was significantly increased as compared with PmCQ2 and C-*qseC* (Figure 1G). QseC positively regulates oxidative stress, osmotic pressure, and heat shock resistance in *Glaesserella parasuis* [19]. However, QseC in *P. multocida* was involved in the negative regulation of oxidative stress (Figure 2A) and osmotic pressure resistance (Figure 2B), opposite to *G. parasuis*.

### Role of QseC in *P. multocida* virulence

**Figure 3:**
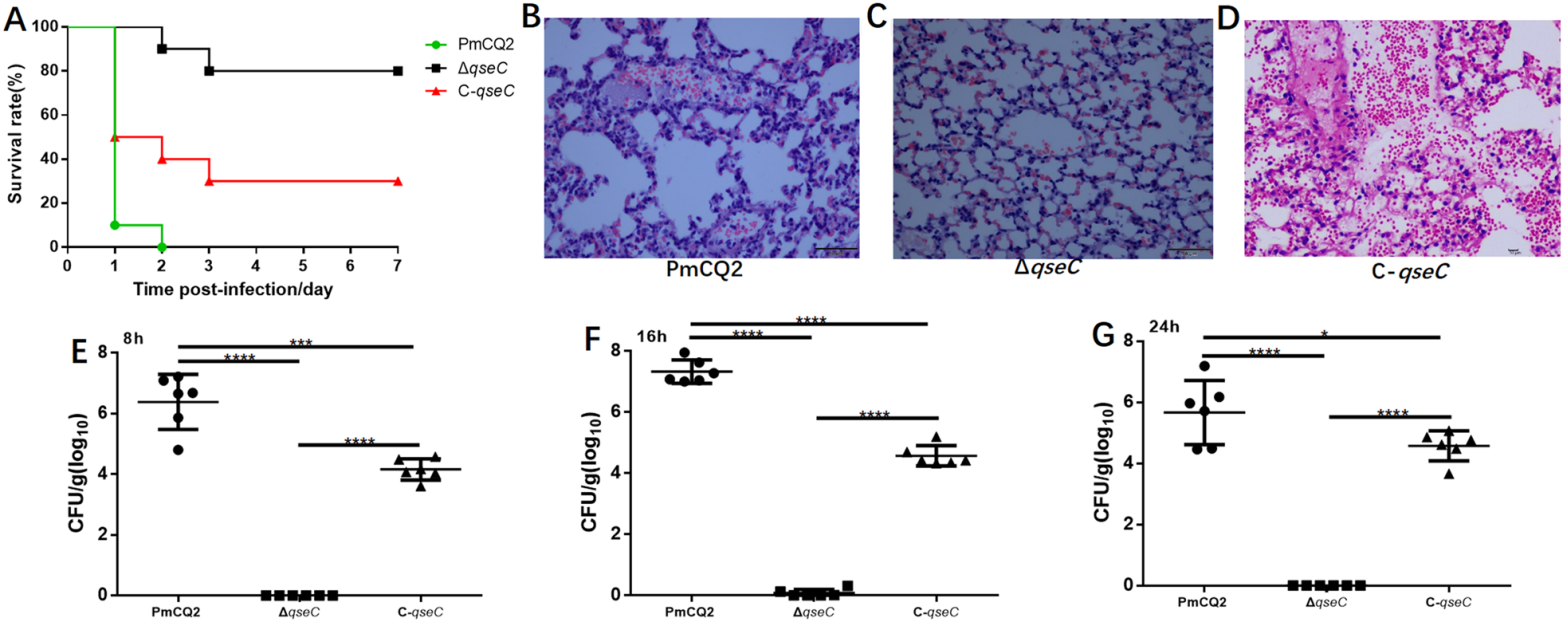
**Pathogenicity analyses**. **A** Survival rates of mice infected with wild strain PmCQ2, Δ*qseC* and C-*qseC*, respectively, *n* = 10 mice/group. **B**–**D** Histopathology in lung of mice 24 h after infection with PmCQ2, Δ*qseC* and C-*qseC*, respectively. **B** Serious dilation and congestion in veins and alveolar wall capillaries, with inflammatory cell infiltration; **C** Slight dilation and congestion in alveolar wall capillaries, with a small number of red blood cells in some alveolar cavities; **D** Hyperemia and bleeding, with large number of red blood cells and cellulose exudation in the alveolar cavity. **E**–**G** Bacterial loads in the mice lungs at 8, 16, and 24 h after infection with PmCQ2, Δ*qseC* and C-*qseC*, respectively. Panels **B**–**G**: values are expressed as the mean ± SD, *n* = 6. * *P* < 0.05; *** *P* < 0.001; **** *P* < 0.0001.

QseC can regulate virulence in many bacterial pathogens. To investigated its effect on the virulence in *P. multocida*, mice were injected with PmCQ2, Δ*qseC*, and C-*qseC* (3.48 × 10^5^ CFU), respectively. The survival rate of mice infected with Δ*qseC* was significantly higher than that of mice infected with PmCQ2 or C-*qseC* (Figure 3A). Compared with WT and C-*qseC*, the *qseC* mutant induced a weak inflammatory response in lung of mice (Figures 3B and C), and the bacterial loads in the lungs of mice infected with *qseC* mutant were significantly lower than that in PmCQ2 and C-*qseC* infection (Figures 3D–F). To further quantify the decrease of virulence in *qseC* mutant, 50% lethal dose assays were conducted. The LD_50_ of Δ*qseC* via intraperitoneal route was 5.28 × 10^7^ CFU, which was 5.28 × 10^7^ fold higher than that of PmCQ2 (intraperitoneal route: LD_50_ ≈ 1 CFU) [28], and 2.1 × 10^3^ fold higher than that of C-*qseC* (intraperitoneal route: LD_50_ = 2.48 × 10^4^ CFU) (Table 3).

**Table 3:** **LD**_**50**_ **calculations.**

Δ*qseC*	Infection dose (CFU)	3.72 × 10^8^	4.46 × 10^7^	4.46 × 10^6^	4.46 × 10^4^
	Death/Total mice	9/10	3/10	1/10	2/10
	LD_50_ = 5.28 × 10^7^ CFU				
C-*qseC*	Infection dose (CFU)	3.80 × 10^6^	3.80 × 10^4^	3.80 × 10^3^	3.80 × 10^2^
	Death/Total mice	9/10	6/10	4/10	0/10
	LD_50_ = 2.48 × 10^4^ CFU				

### QseC regulates *P. multocida* gene expression

**Figure 4:**
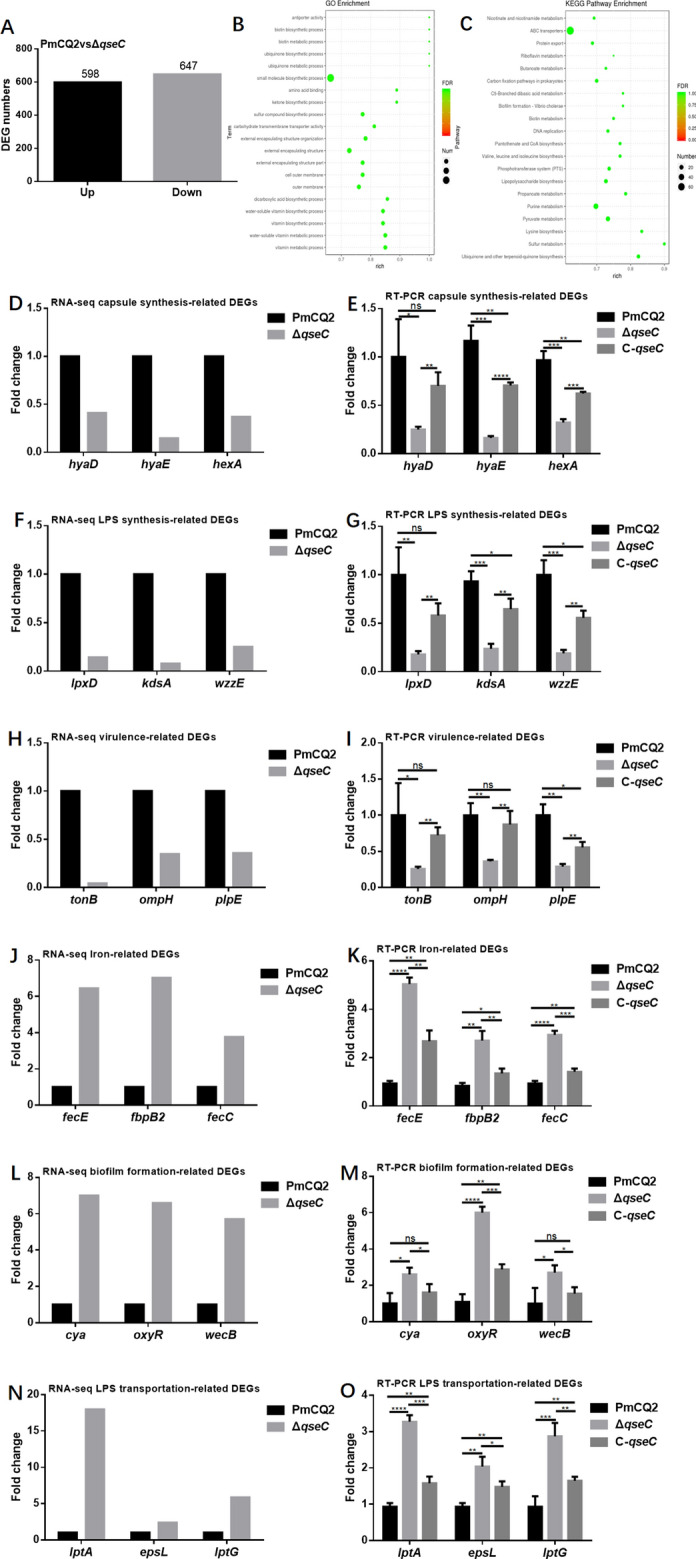
**Transcriptome analysis of wild-type PmCQ2 and Δ*qseC*** **in vitro**. **A** The up/down-regulated DEGs (FC ≥ 1.5) of PmCQ2 and Δ*qseC* in vitro. **B**, **C** GO and KEGG pathway analysis of PmCQ2 and Δ*qseC*. **D**, **E** Capsular synthesis-related DEGs (FC ≥ 1.5) in RNA-seq and in RT-PCR (*n* = 3). **F**, **G** LPS synthesis related DEGs (FC ≥ 1.5) in RNA-seq and in RT-PCR (*n* = 3). **H**, **I** Virulence-related DEGs (FC ≥ 1.5) in RNA-seq and in RT-PCR (*n* = 3). **J**, **K** Iron-related DEGs (FC ≥ 1.5) in RNA-seq and in RT-PCR (*n* = 3). **L**, **M** Biofilm formation-related DEGs (FC ≥ 1.5) in RNA-seq and in RT-PCR (*n* = 3). **N**, **O** LPS transportation-related DEGs (FC ≥ 1.5) in RNA-seq and in RT-PCR (*n* = 3). Panels (**E**, **G**, **I**, **K**, **M**, **O**) were pooled from three independent experiments with 3 replicates per group and analyzed with multiple comparative analysis. All data are expressed as mean ± SD. * *P* < 0.05; ** *P* < 0.01; *** *P* < 0.001, **** *P* < 0.0001; ns means not significant.

**Figure 5:**
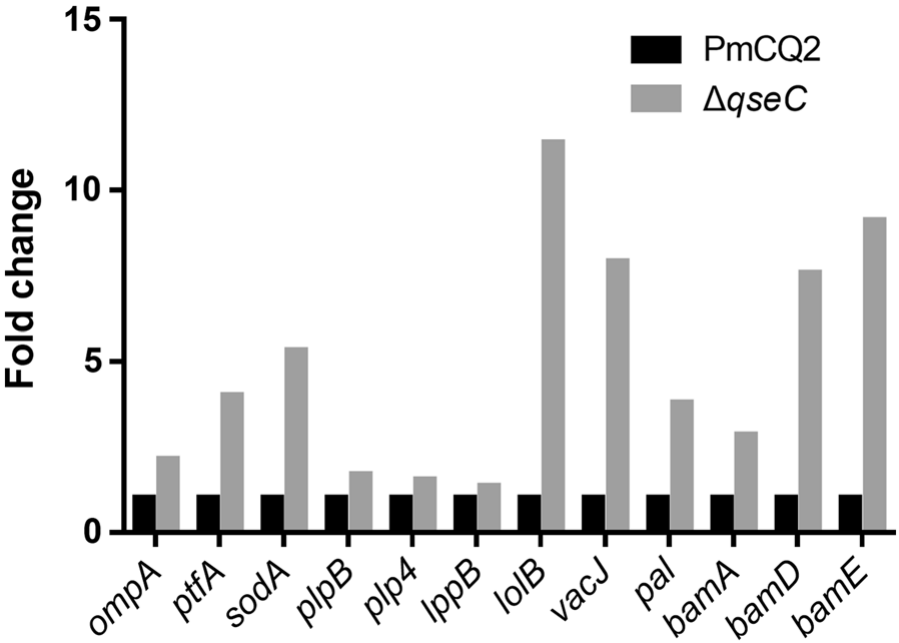
**Significantly up-regulated genes encoding for immuno-protection in Δ*qseC* based on transcriptome sequencing**.

We next conducted transcriptome sequencing of wild-type strain and Δ*qseC* strain (NCBI’s Sequence Read Archive (SRA) database accession numbers are PRJNA629381 and PRJNA687922). A total of 1245 differentially expressed genes (DEGs) between PmCQ2 and Δ*qseC* (Fold change ≥ 1.5) were annotated (Figure 4A). GO and KEGG pathway analysis demonstrated that the top DEGs are involved in outer membrane, ABC transporters, biofilm formation, lipopolysaccharide biosynthesis, and amino acid biosynthesis (Figures 4B and C). Consistent with changes in biological characteristics, genes for capsule synthesis (Figures 4D and E), LPS synthesis (Figures 4F and G) and virulence (Figures 4H and I) were significantly down-regulated in Δ*qseC*. However, most genes involved in iron utilization/transport (Figures 4J and K), biofilm formation (Figures 4L and M), LPS transport (Figures 4N and O) and immune protection (Figure 5) were significantly up-regulated.

### Δ*qseC* stimulates a stronger antibody response

**Figure 6:**
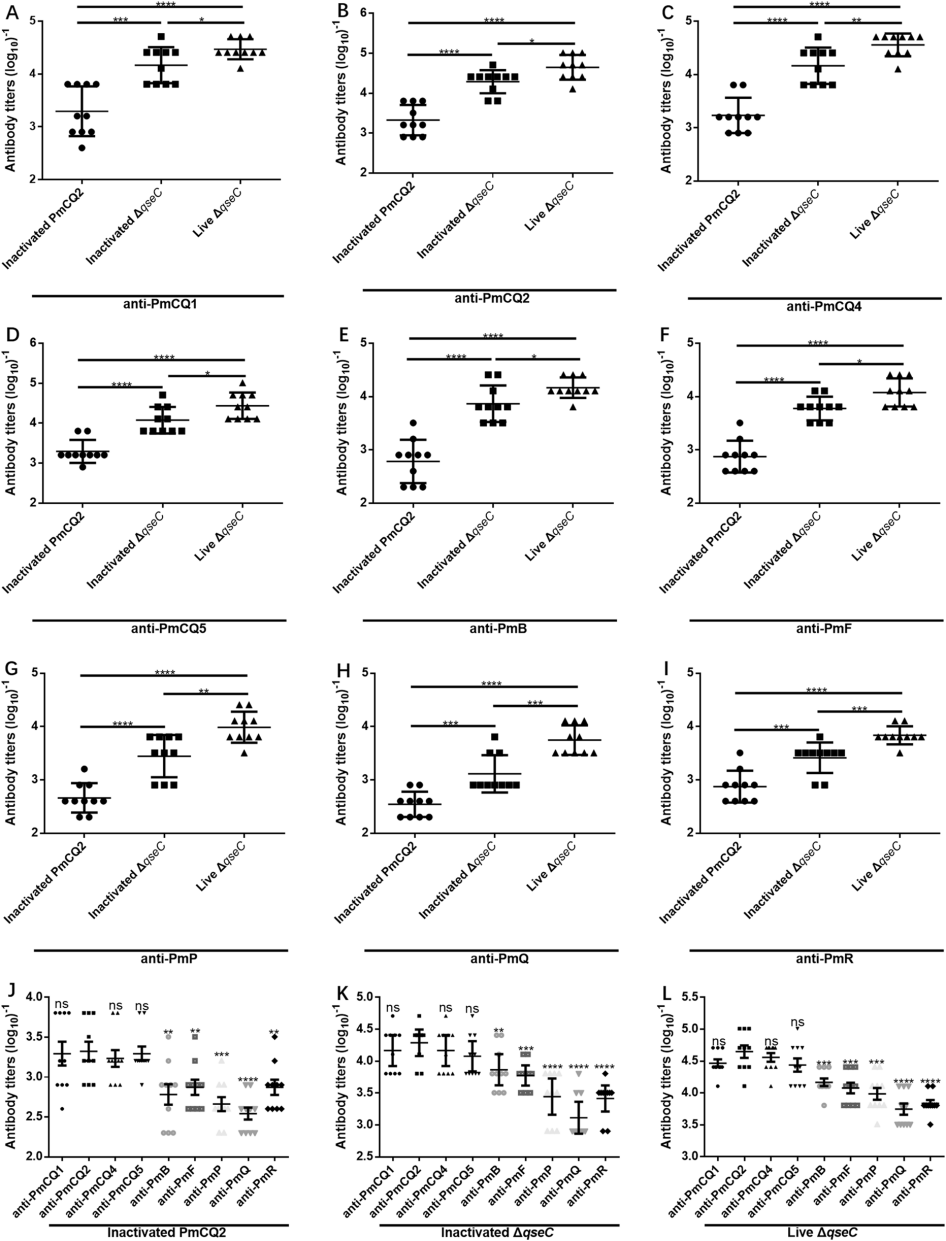
**Serum IgG antibody titers. Mice were immunized with inactivated PmCQ2, inactivated Δ*qseC* and live Δ*qseC*, respectively**. Blood was collected via the tail vein route at 21-day after immunization for serum isolation. The serum antibodies against **A** PmCQ1, **B** PmCQ2, **C** PmCQ4, **D** PmCQ5, **E** PmB, **F** PmF, **G** PmP, **H** PmQ, **I** PmR were detected. The IgG antibody titers of mice immunized with **J** inactivated PmCQ2, **K** inactivated Δ*qseC*, **L** live Δ*qseC* that against different *P. multocida* strains were analyzed. All data are expressed as mean ± SD, *n* = 10 mice/group. * *P* < 0.05; ** *P* < 0.01; *** *P* < 0.001; **** *P* < 0.0001; ns means no significant.

To explore the effect of *qseC* deletion on antibody production, the serum IgG titers of the mice immunized with inactivated PmCQ2, inactivated Δ*qseC* and live Δ*qseC* were measured by using ELISA. The antibody titers of Δ*qseC* were significantly higher than that of PmCQ2 (Figures 6A–I), and compared with inactivated PmCQ2 and inactivated Δ*qseC*, the live Δ*qseC* immunization generated much higher antibody levels against *P. multocida* strains of broad serotype (Figures 6A–I). The titers of serum antibody in immunized mice that against bovine capsular serotype A strains were significantly higher than that against bovine capsular serotype B and F and other animal *P. multocida* capsular serotype A strains (Figures 6J–L). The results indicate that live Δ*qseC* can induce much higher cross-reactive antibodies than inactivated PmCQ2 and inactivated Δ*qseC*.

### Δ*qseC* cross-protects against other serotypes

**Figure 7:**
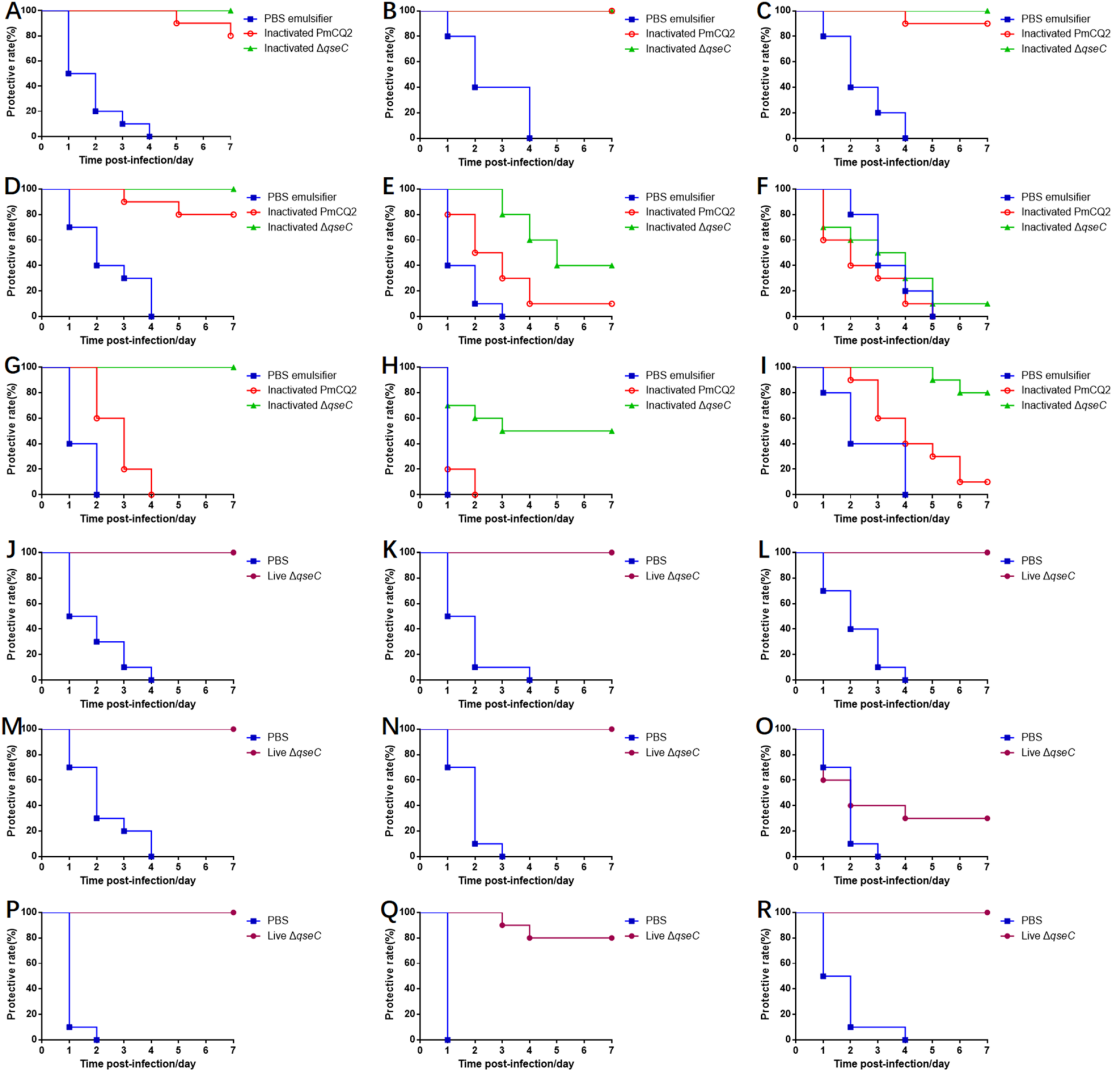
**Immune protection against** ***P. multocida* serotypes induced by live Δ*qseC,* inactivated PmCQ2 and inactivated Δ*qseC*, respectively.** The survival rates of mice subcutaneously immunized with inactivated PmCQ2, inactivated Δ*qseC*, and PBS emulsifier and intramuscularly challenged with **A** PmCQ1, **B** PmCQ2, **C** PmCQ4, **D** PmCQ5, **E** PmB, **F** PmF, **G** PmP, **H** PmQ, and **I** PmR, respectively. The survival rates of mice intramuscularly immunized with live Δ*qseC* and PBS for 21 days and intramuscularly challenged with **J** PmCQ1, **K** PmCQ2, **L** PmCQ4, **M** PmCQ5, **N** PmB, **O** PmF, **P** PmP, **Q** PmQ, and **R** PmR, respectively.

The cross-protection of Δ*qseC* against other *P. multocida* serotypes were investigated. Kunming mice were intramuscularly inoculated with live Δ*qseC*, inactivated Δ*qseC* and inactivated PmCQ2, respectively. At day-21 after primary immunization, mice were challenged with 9 strains of *P. multocida*, respectively (Table 2). Live and inactivated Δ*qseC* both presented strong cross-protection to mice against multiple *P. multocida* strains (Figures 7A–R). However, inactivated PmCQ2 presented no cross-protection to mice against bovine *P. multocida* serotypes B and F, and rabbit, avian and porcine *P. multocida* serotype A (Figures 7G–I). Immunization with live Δ*qseC* presented 100% protection to mice against infection by bovine *P. multocida* capsular serotype A (Figures 7J–M) and serotype B (Figure 7N), porcine *P. multocida* capsular serotype A (Figure 7P) and rabbit *P. multocida* capsular serotype A (Figure 7R). However, live Δ*qseC* only presented 30% protection to bovine *P. multocida* capsular serotype F infection (Figure 7O), and 80% protection to avian *P. multocida* capsular serotype A infection (Figure 7Q). Even though inactivated Δ*qseC* also presented a good cross-protection, the live Δ*qseC* showed better (Figures 7A–R). The results indicate that live Δ*qseC* has potential to develop as an attenuated vaccine against infection by *P. multocida* strains of homologous and heterologous serotypes.

## Discussion

In the present study, we showed that QseC regulates capsular production, biofilm formation, stress resistance, and virulence in *P. multocida*, consistent with previous reports for other bacterial pathogens. We also showed that deleting *qseC* might provide a means by which to develop a cross-protective *P. multocida* vaccine.

At present, a few commercial *P. multocida* vaccines provide limited immune protection to domestic animals against infection by *P. multocida* of homologous serotypes [29]. The lack of a universal vaccine has brought challenges to prevent *P. multocida* infections. Although antibiotics can be used to control *P. multocida*, however, with the extensive use of antibiotics, many strains are now drug-resistant [30]. The previous studies in the discovery of cross protective antigens such as PlpB [31], PlpE [32] and PmCQ2_2g0128 [33] in *P. multocida* provides a basis for the research of cross-protective vaccine against *P. multocida*.

Deletion of the *aroA* gene in *P. multocide* P-1059 strain (serotype A:3) promoted strain cross-protection against infection by *P. multocide* serotype A:1 or A:4 [20], which in part motivated this study. We speculated that the absence of QseC in *P. multocida* could also lead to the down-regulation of virulence and promote the cross-immune protection characteristics of strains.

QseC belongs to the QseBC quorum sensing system, which regulates virulence gene expression in many pathogenic bacteria [34, 35]. As one of two-component regulators, QseC can activate the downstream receptor QscB to facilitate the expression of associated genes. We observed that the expression of *qseB* was promoted by deleting *qseC* (Additional file 3). There are two potential transcriptional start sites in the *qseBC* promoter [12]; deleting *qseC* abolishes only the first transcription initiation site. The absence of QseC in *E. coli* leads to activation of QseB by a sensor kinase PmrB [36]. In *P. multocida*, there may also exist a sensor like PmrB to activate QseB in the absence of QseC.

To our knowledge, this is the first report that *qseC* deletion in bacteria can regulate strain cross immune protection. Recombinant QseC can induce host innate immunity, which may reduce the virulence gene expression of avian pathogenic *E. coli* [37], suggesting QseC has potential as a vaccine candidate against bacterial pathogen infections. The development of vaccine combined with rQseC and *qseC* mutant infection may be a next research orientation.

In the present study, deleting *qseC* resulted in a significant decrease in the production of capsular polysaccharides; however, transcriptome analysis showed that the expression levels of only a few genes related to capsular polysaccharide synthesis were down-regulated, while the expression of most genes were significantly up-regulated. Although some reports suggest that the presence of the capsule can mask the relevant immune antigens, thus affecting antigen presentation, it does not mean the absence of capsule will certainly enhance the induction of immune protection by the strain. Deleting *cexA* or *bcbH* can lead to capsule loss of *P. multocida*, but the immune protection characteristics of the deletion strains were significantly different [38]. We observed a similar phenotype in studies of a Pm0442 deletion [22]. Therefore, the capsule deficiency in *P. multocida* is likely not the key to increased immune protection in strains.

The serum antibody titers of mice immunized with Δ*qseC* were significantly higher than after immunization with the wild type strain. We speculate that there may be some cross-protective antigens up-regulated in Δ*qseC*. Transcriptome sequence showed that many genes encoding outer membrane proteins (*plpB*, *plp4*, *lppB*, *lolB*, *vacJ*, *pal*, *bamA*, *bamD*, and *bamE*) were up-regulated (Figure 4); some of these genes are associated with immune protection or cross-protection in pathogens [31, 39–41]. Even though the general virulence was decreased in Δ*qseC*, we also found that the expression of some virulence-related genes such as *ompA*, *ptfA*, and *sodA* were up-regulated.

In summary, our study demonstrates that QseC mediates capsule production, biofilm formation, resistance to environmental stress, and virulence in *P. multocida*. Deleting *qseC* promotes *P. multocida* cross- immune protection against infection by *P. multocida* strains of homologous and heterologous serotypes.

## Supplementary Information

**Additional file 1**: **qRT-PCR primers**.

**Additional file 2**: **Phylogenetic tree constructed based on the complete QseC amino acid sequences**. The phylogenetic tree of QseC was constructed by employing the Neighbor-Joining method in MEGA X. The complete QseC amino acid sequences from 264 strains of Pasteurella multocida, 2 strains of Salmonella enterica, 2 strains of *Escherichia coli*, and 1 strain of following bacteria including *Pasteurella canis*, *Pasteurella dagmatis*, *Pasteurella oralis*, *Pasteurella aerogenes*, *Moraxella* sp., *Frederiksenia canicola*, *Necropsobacter massiliensis*, *Caviibacterium falvescens*, *Haemophilus felis*, were obtained from GenBank.

**Additional file 3: Relative expression of qseB in PmCQ2 and ΔqseC. Overnight cultured PmCQ2 (1 × 10**^**8**^ **CFU) and ΔqseC (1 × 10** ^**8**^ **CFU) were inoculated into 5 mL Martin broth medium, respectively, incubated at 37 °C with shaking at 200 rpm**. Bacterial cells were collected at 3, 6, and 9 h for RNA extraction, A-C qseB gene expression in PmCQ2 and ΔqseC. Panels (A-C): all values are expressed as mean ± SD, n = 5. ** P < 0.01, *** P < 0.001.

## Data Availability

All data generated or analyzed during this study are included in this article.

## Abbreviations

Qse: Quorum sensing
Omp: Outer membrane protein
LPS: Lipopolysaccharide
DEGs: Differentially expressed genes
AHL: Acyl-homoserine lactone
AI-2: Autoinducers 2
AI-3: Autoinducers 3
