# Supplementary material for: Deleting qseC downregulates virulence and promotes cross-protection in Pasteurella multocida
Source: Vet Res. 2021 Nov 20;52:140. doi: 10.1186/s13567-021-01009-6 (PMC8605557; doi:10.1186/s13567-021-01009-6)
Supplement: Supplementary file 1 — Additional file 1: qRT-PCR primers. [file 13567_2021_1009_MOESM1_ESM.docx]

**Additional file 1 qRT-PCR primers.**

| **Primer name** | **Sequence（5’-3’）** | **Product size (bp)** |
| --- | --- | --- |
| *qseC*-F/*qseC*-R | TCACGTTATCGTTGACCGCA / AGGCCAAACGCTGAGCTAA T | 126 |
| *hyaD*-F/*hyaD*-R | GGCGGGTAATGTTGCTTTCG / TCCACATCTTCTCCACCCCA | 90 |
| *hyaE*-F/*hyaE*-R | GCTAAACCACGCCTTTACGG / ACTCGTGAATTGGTGGGAGG | 196 |
| *hexA*-F/*hexA*-R | AATCGTACGGGGTGGCATAC / AAGGGTTGATTTCCCTGCCC | 105 |
| *lpxD*-F/*lpxD*-R | TCCATATTGGGACCGGGACT/ CGAAGAGTACACGCCAGGTT | 188 |
| *kdsA*-F/*kdsA*-R | AACCGATTTAGTGGCAGCGA / ATTCCCCATTTGACCGGGAC | 88 |
| *wzzE*-F/*wzzE*-R | TTGGGGACTACGTTGCTTGG / CACATGTGCTTCTGCAAGCC | 84 |
| *tonB*-F/*tonB*-R | TAATGGGCTCACAATGGCGT / TGCTGCCAGATCGGTCTTTT | 140 |
| *ompH*-F/ *ompH*-R | GCTTGGGGTGCTGACAATAA / TTATTCACGCCATAACCCGC | 248 |
| *plpE*-F/ *plpE*-R | TGGAGCTGAATTTTATCAACGCA / TTTTCAGCTTTTCCTACACCAACA | 70 |
| *fbpB2*-F/*fbpB2*-R | GCCCACGATCCAGTCAAAGA / TTTCGTCCGCTCAATGACCA | 184 |
| *fecC*-F/*fecC*-R | CGAAGGCGAGGCTAAAGGAT / TACCCAACCCCGATTTCAGC | 189 |
| *fecE*-F/*fecE*-R | GTTCGGTCATCACCTCGTGT / GATGTCAATGATGCGACGCC | 155 |
| *wecB*-F/*wecB*-R | TCGGGTTCTTGGATGTGTCG / GTGCACACCGCGAAGAAAAT | 122 |
| *cya*-F/*cya*-R | GTGACAAAATGCCGCAAGGT / AACGTTCAAAGACGGGTGGT | 198 |
| *oxyR*-F/*oxyR*-R | ATCACCCTTGGGCGAATGAG / GCTCCTGCACTGAAGCAGTA | 124 |
| *lptA*-F/*lptA*-R | TTGGCATGCCCTTCTACAGG / GGACAACAGCGTGGTAACCT | 192 |
| *epsl*-F/*epsl*-R | CTGCTGTCCTTCATTGTGCG / TGATTTCAGCCACTGCGCTA | 163 |
| *lptG*-F/*lptG*-R | GATGGCTGCTTTATTGGGCG / AGATGCCTGCATCACAACCA | 79 |
