# Supplementary figures and images for: Deleting qseC downregulates virulence and promotes cross-protection in Pasteurella multocida
Source: Vet Res. 2021 Nov 20;52:140. doi: 10.1186/s13567-021-01009-6 (PMC8605557; doi:10.1186/s13567-021-01009-6)

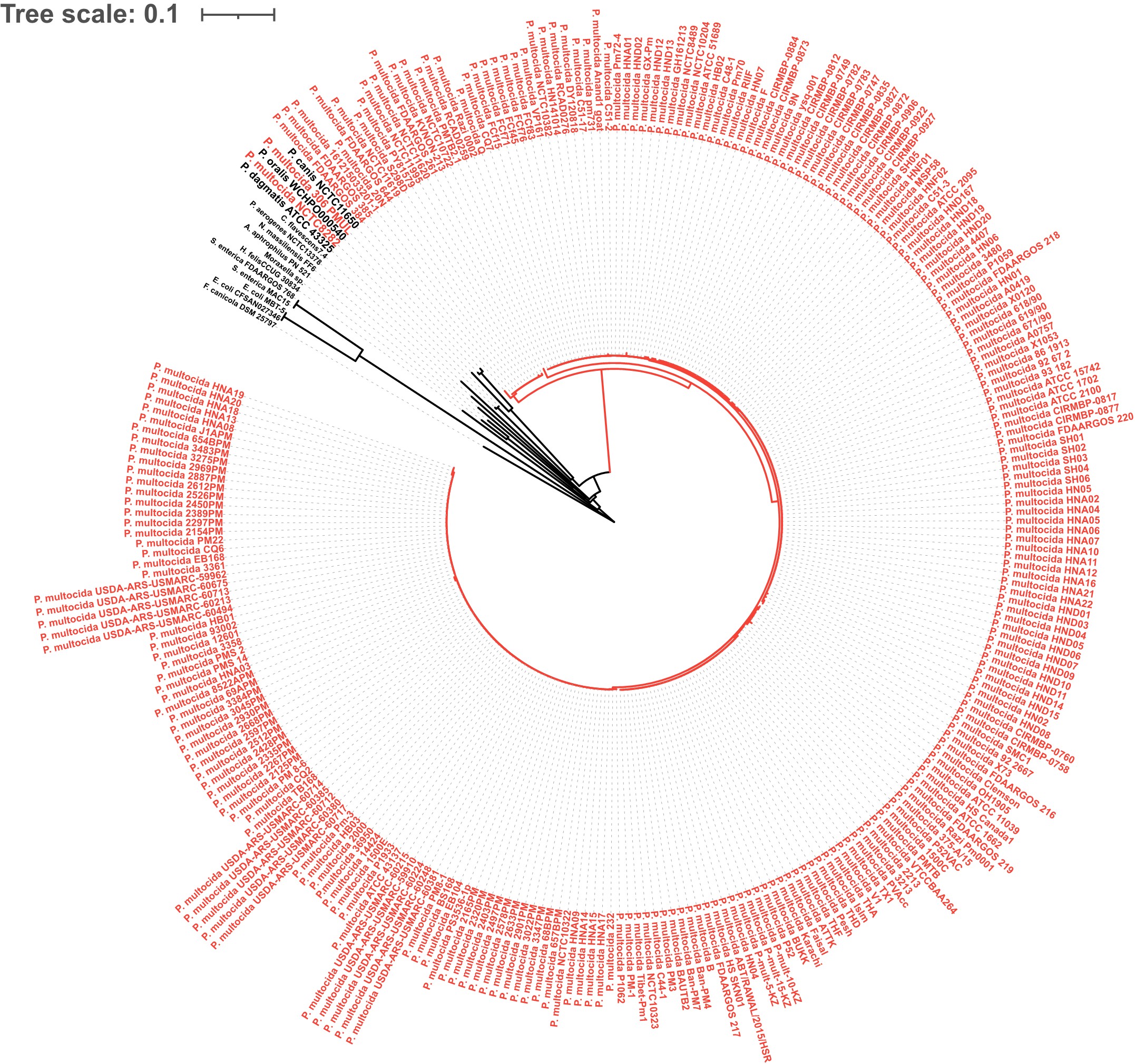

Supplement: Supplementary file 2 — Additional file 2: Phylogenetic tree constructed based on the complete QseC amino acid sequences. The phylogenetic tree of QseC was constructed by employing the Neighbor-Joining method in MEGA X. The complete QseC amino acid sequences from 264 strains of Pasteurella multocida, 2 strains of Salmonella enterica, 2 strains of Escherichia coli, and 1 strain of following bacteria including Pasteurella canis, Pasteurella dagmatis, Pasteurella oralis, Pasteurella aerogenes, Moraxella sp., Frederiksenia canicola, Necropsobacter massiliensis, Caviibacterium falvescens, Haemophilus felis, were obtained from GenBank. [file 13567_2021_1009_MOESM2_ESM.jpg]

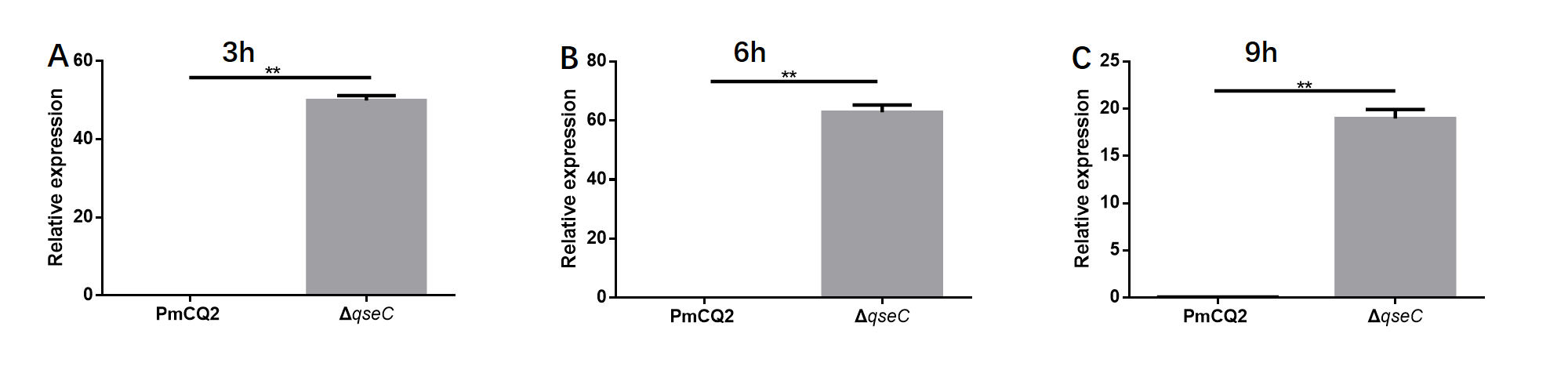

Supplement: Supplementary file 3 — Additional file 3: Relative expression of qseB in PmCQ2 and ΔqseC. Overnight cultured PmCQ2 (1 × 10^8^ CFU) and ΔqseC (1 × 10 8 CFU) were inoculated into 5 mL Martin broth medium, respectively, incubated at 37 °C with shaking at 200 rpm. Bacterial cells were collected at 3, 6, and 9 h for RNA extraction, A-C qseB gene expression in PmCQ2 and ΔqseC. Panels (A-C): all values are expressed as mean ± SD, n = 5. ** P < 0.01, *** P < 0.001. [file 13567_2021_1009_MOESM3_ESM.tif]
